# Persistent Median Artery and Carpal Tunnel Syndrome: A Retrospective Study

**DOI:** 10.1055/s-0044-1785657

**Published:** 2024-05-13

**Authors:** Yago de Andrade Fonseca Felix, Vitor Henrique de Lima Pistilli, Luis Guilherme Rosifini Alves Rezende, Filipe Jun Shimaoka, Luiz Garcia Mandarano-Filho, Nilton Mazzer

**Affiliations:** 1 Departamento de Ortopedia e Anestesiologia, Hospital das Clínicas, Faculdade de Medicina de Ribeirão Preto, Universidade de São Paulo, Ribeirão Preto, SP, Brasil

**Keywords:** carpal tunnel syndrome, median artery, median nerve, median neuropathy

## Abstract

**Objective** This study presents a retrospective study of persistent median artery associated with carpal tunnel syndrome (CTS).

**Methods** A retrospective study of the persistent median artery and CTS. Exclusion criteria were patients who did not present persistent median artery, those who were diabetic, or had rheumatoid diseases, and those who decided not to do the surgery. Only 25 patients were eligible for this retrospective study.

**Results** Median artery thrombosis had statistical differences considering the variables sex ( *p* = 0.009), electroneuromyography findings ( *p* = 0.021), profession ( *p* = 0.066), and “total duration since the beginning of the symptoms” ( *p* = 0.055). Thenar muscle atrophy had no statistical differences when compared to the variables. Bifid median nerve had statistical differences when compared to provocative tests ( *p* = 0.013), frequency of symptoms ( *p* = 0.001), and age ( *p* = 0.028).

**Conclusion** Although uncommon, the persistent median artery should be considered a differential diagnosis for CTS. Ultrasonography is a reliable method to predict carpal tunnel anatomy. Late onset and symptoms could influence artery thrombosis and worsen the symptoms.

## Introduction

Carpal tunnel syndrome (CTS) is a compressive disorder of the median nerve at the wrist level that affects approximately 4% of the general population, being the most common compressive neuropathy of the upper limb. 1 2 This pathology was initially described by compression caused by a distal radius fracture by Paget in 1854. 3 4 5 The median nerve progresses to the wrist level adjacent to nine tendons in the carpal tunnel, an inextensible osteofibrous canal. Patients complain of pain and paresthesia, worst at night. Weakness of thenar muscles is common and could evolute to thenar muscle atrophy (TMA). 5 6

Several causes could compress the median nerve, either idiopathic or secondary. 6 Among the secondary causes are endocrine-metabolic alterations (pregnan, diabetes), fractures and dislocations, tumors (ganglions, lipomas), systemic causes (rheumatoid), anatomical variations and persistence of the Median artery. 2 6 7

The persistent Median artery is present in approximately 0.9 to 16% of the population. 7 It is present in the embryonic period, being the dominant bloodstream in the embryonic hand, helping the development of the arteries of the upper limb and involuting from the 8th week of pregnancy. In this phase, the ulnar and radial arteries formation begins, which will become the main vascular supply of the distal territory of the upper limb. 7 8 9

The persistence of the Median artery in the fetal period could contribute to the development of the superficial palmar arch, which may be more exuberant than usual. When traveling through the carpal tunnel together with the Median nerve, although rare, it can trigger carpal tunnel syndrome. 10

Median artery persistence can occur in two patterns: antebrachial and palmar. 11 In the first, the Median artery ends as a muscular branch, not reaching the wrist with no pathogenesis. In the second type, the Median artery extends to the palmar territory, crossing the carpal tunnel, sometimes participating in the formation of the superficial palmar arch, and becoming a possible cause for carpal tunnel syndrome. 2 8 11 12

## Methods

This study was approved by our institutional review board (CAAE-45542621.2.0000.5440). The patient and his family were informed that data from the case would be submitted for publication and gave their consent.

A retrospective study reviewed the medical records of patients who presented carpal tunnel syndrome associated with persistent median artery submitted to decompression surgery in a tertiary hospital.

**Fig. 1:**
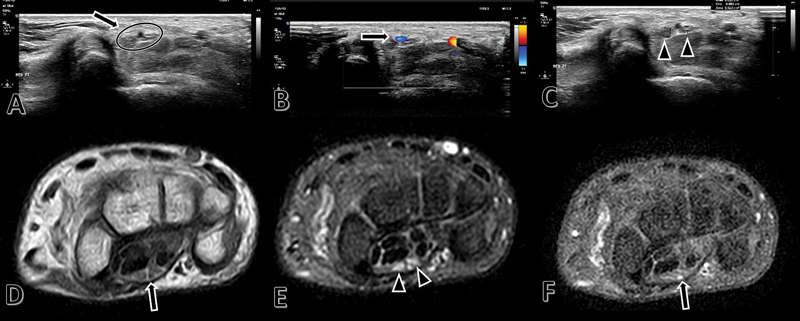
Ultrasonography. **A** : Median nerve (arrow, circle). **B** : Persistent median artery (arrow). **C** : Bifid median nerve (arrowheads). **D, E, F** : MRI scan. **D** : Median nerve (arrow). **E** : Bifid median nerve (arrowheads). **F** : Persistent median artery (arrow).

There were 1,276 patients with carpal tunnel syndrome submitted to decompression between 2012 to 2020. Inclusion criteria were patients who presented persistent Median artery and carpal tunnel syndrome from 2012 to 2020, being 38 patients. Exclusion criteria were patients who did not present the persistent median artery, diabetic, rheumatoid diseases and those who decided not to do the surgery. Only 25 patients were eligible for this retrospective study. We used the data present in the medical records and imaging exams, such as ultrasound and MRI ( Fig. 1 ).

The mean age was 41 years (ranging from 18–73 years), and there were 17 females and 6 males. Furthermore, 24 patients were righthanded. The profession and variables of the participants are presented in Table 1 . The provocative tests (Durkan and Phalen) were present in all the patients during physical evaluation. However, 11 patients present numbness during physical evaluation. There were 5 patients who reported intermittent symptoms, and 20 were continuous. All the patients complained about weakness; however, only four patients presented with TMA. The preoperative protocol includes ultrasonography (Doppler) to measure the carpal tunnel and the median nerve. Electroneuromyography (EMG) was performed for all patients.

**Table 1:** Cases and variables

	Sex	Age (years)	Ethinicity	Dominance	AFW	Profession	Comorbidities	EMG	PT	FS	TMA	BMN	MAT	DSUS (months)
**Case 1**	F	60	AA	R	R	Kitchen	ASH	Y	DP	C	N	Y	N	50
**Case 2**	F	36	W	R	R	Manicure	N	Y	DPN	C	N	Y	N	49
**Case 3**	F	40	AA	R	R	Maid	ASH	N	DPN	C	Y	Y	N	36
**Case 4**	F	40	AA	R	L	Maid	ASH	N	DPN	C	N	Y	N	52
**Case 5**	F	28	AA	R	R	Maid	N	N	DPN	C	N	Y	N	216
**Case 6**	F	26	W	R	L	Maid	N	N	DPN	C	N	Y	N	204
**Case 7**	F	31	W	R	R	Maid	N	N	DPN	C	Y	Y	N	130
**Case 8**	M	45	W	R	R	GS	ASH	N	DPN	C	N	Y	N	133
**Case 9**	M	49	W	L	L	Maid	ASH	N	DPN	C	N	Y	N	131
**Case 10**	F	38	AA	R	L	Driver	N	N	DPN	C	Y	Y	N	118
**Case 11**	F	33	W	R	R	GS	N	N	DPN	C	N	Y	N	83
**Case 12**	F	35	W	R	L	Maid	N	N	DPN	C	N	Y	N	72
**Case 13**	M	44	W	R	R	Nurse	N	N	DP	C	Y	Y	S	41
**Case 14**	F	26	AA	R	L	Nurse	N	Y	DP	C	N	Y	S	42
**Case 15**	M	18	W	R	R	GS	N	Y	DP	C	N	Y	S	33
**Case 16**	M	45	W	R	R	BP	N	Y	DP	C	N	Y	S	48
**Case 17**	M	35	AA	R	L	BP	N	Y	DP	C	N	Y	S	47
**Case 18**	M	44	W	R	R	Professor	N	Y	DP	C	N	Y	S	52
**Case 19**	F	38	W	R	R	Professor	N	N	DP	C	N	N	S	55
**Case 20**	F	64	W	R	R	BP	N	Y	DP	I	N	N	N	51
**Case 21**	F	45	W	R	R	Kitchen	N	N	DP	I	N	N	N	49
**Case 22**	F	30	AA	R	L	GS	N	N	DP	I	N	N	N	47
**Case 23**	F	54	W	R	R	Dressmaker	ASH	N	DP	I	N	N	N	39
**Case 24**	F	48	AA	R	R	Kitchen	ASH	N	DP	I	N	Y	N	44
**Case 25**	M	73	W	R	L	Professor	ASH	Y	DP	C	N	N	N	91

**Abbreviations:** AA, afro american; AFW, affected wrist side; ASH, arterial systemic hypertension; BMN, bifid median nerve; BP, businessperson; C, continuous; DP, Durkan-Phalen; DPN, Durkan-Phalen-numbness; DSUS, duration of symptoms until surgery; EMG, electroneuromyography; F, female; FS, frequency of symptoms; GS, general services; I, intermittent; L, left; M, male; MAT, median artery thrombosis; TMA, thenar muscle atrophy; N, No; PT, provocative tests; R, right; W, white; Y, yes.

**Fig. 2:**
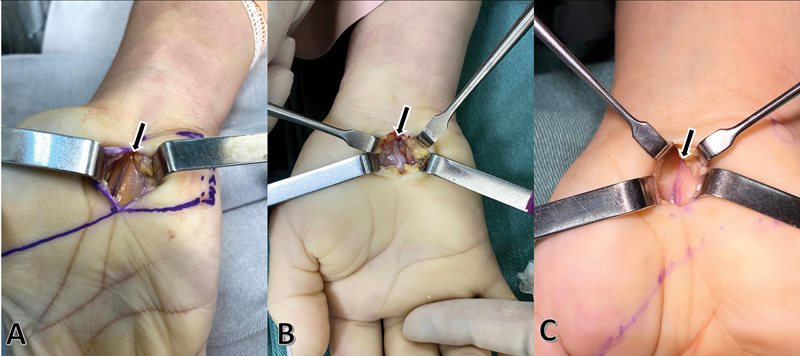
The operative aspect of the persistent median artery (arrow).

The patients were submitted to surgical decompression of the median nerve at the wrist level, 17 on the right wrist and eight on the left wrist ( Fig. 2 ). There were 19 patients who presented with bifid median nerve (BMN), as observed in the ultrasonography. There were seven TMAs during surgical evaluation. No complications were reported (infection, wound complications, recurrence). The mean follow-up was 76.5 months (ranging from 33 to 216), with no recurrence reported yet.

The Student t-test and Chi-squared tests were used to analyze the variables. A two-tailed p-value of less than 0.05 was considered statistically significant. All analyses were performed using the Statistical Package Social Sciences (SPSS, IBM Corp., Armonk, NY, USA) for Os X, version 22.0.

## Results

There were no statistical differences between the variable dominance (Chi-square: *p* = 0.524; Fisher: *p* = 1.000), affected wrist side (Chi-square: *p* = 0.819; Fisher: *p* = 1.000), ethnicity (Chi-square: *p* = 0.856; Fisher: *p* = 1.001), BMN (Chi-square: *p* = 0.478; Fisher: *p* = 0.637), TMA (Chi-square: *p* = 0.884; Fisher: *p* = 1.000), frequency of symptoms (Chi-square: *p* = 0.119; Fisher: *p* = 0.274) and median artery thrombosis (MAT).

There were statistical differences for MAT considering the variables sex (Chi-square: *p* = 0.009; Fisher: *p* = 0.023), EMG findings (Chi-square: *p* = 0.021; Fisher: *p* = 0.061), comorbidities (Chi-square: *p* = 0.032; Fisher: *p* = 0.057), provocative test (Chi-square: *p* = 0.006; Fisher: *p* = 0.008), and profession (Chi-square: *p* = 0.066), with maids having a higher risk of MAT.

There were statistical differences between the variables “total duration since the beginning of the symptoms” (ANOVA: *p* = 0.055, Levene test: 0.003) and MAT. There were no statistical differences in the variable age (ANOVA: *p* = 0.197, Levene: 0.461) and MAT.

There were no statistical differences between the variable sex (Chi-square: *p* = 0.743; Fisher: *p* = 1.000), dominance (Chi-square: *p* = 0.656; Fisher: *p* = 1.000), affected wrist side (Chi-square: *p* = 0.743; Fisher: *p* = 1.000), ethnicity (Chi-square: *p* = 0.119; Fisher: *p* = 0.292), profession (Chi-square: *p* = 0.222), comorbidities (Chi-square: *p* = 0.743; Fisher: *p* = 1.000), EMG findings (Chi-square: *p* = 0.102; Fisher: *p* = 0.261), provocative tests (Chi-square: *p* = 0.173; Fisher: *p* = 0.303), frequency of symptoms (Chi-square: *p* = 0.275; Fisher: *p* = 0.549), BMN (Chi-square: *p* = 0.220; Fisher: *p* = 0.540) and TMA. There were no statistical differences between the variables “total duration since the beginning of the symptoms” (ANOVA: *p* = 0.844, Levene: 0.811) or age (ANOVA: *p* = 0.644, Levene: 0.111) and TMA.

There were no statistical differences between the variable sex (Chi-square: *p* = 0.356; Fisher: *p* = 0.624), dominance (Chi-square: *p* = 0.566; Fisher: *p* = 1.000), affected wrist side (Chi-square: *p* = 0.356; Fisher: *p* = 0.624), ethnicity (Chi-square: *p* = 0.181; Fisher: *p* = 0.345), profession (Chi-square: *p* = 0.270), comorbidities (Chi-square: *p* = 0.936; Fisher: *p* = 1.014), EMG findings (Chi-square: *p* = 0.876; Fisher: *p* = 1.000). There were statistical differences between the variables provocative tests (Chi-square: *p* = 0.013; Fisher: *p* = 0.020), frequency of symptoms (Chi-square: *p* = 0.001; Fisher: *p* = 0.005), and BMN. There were no statistical differences between the variables “total duration since the beginning of the symptoms” (ANOVA: *p* = 0.251, Levene: 0.018) and BMN. However, there was a statistical difference in the variable age (ANOVA: *p* = 0.028, Levene: 0.089) and BMN.

The results of the variables are shown in Table 2 . All the ultrasonography studies and the operative findings (BMN and persistent median artery) were statistically relevant (Chi-square: *p* = 0.001).

**Table 2:** Results of the studied variables and median artery thrombosis, bifid median nerve, and thenar muscle atrophy

	MAT	BMN	TMA
** Sex ^+^**	0.009	0.356	0.743
** Dominance ^+^**	0.524	0.566	0.656
** AFW ^+^**	0.819	0.356	0.743
** Ethnicity ^+^**	0.856	0.181	0.119
** BMN ^+^**	0.478	X	0.220
** FS ^+^**	0.119	0.001	0.275
** TMA ^+^**	0.884	0.220	X
** EMG findings ^+^**	0.021	0.876	0.102
** Profession ^+^**	0.066	0.270	0.222
** Comorbidities ^+^**	0,032	0.936	0.743
** PT ^+^**	0,006	0.013	0.173
Total duration since the beginning of the symptoms*	0.055	0.251	0.844
Age*	0.197	0.028	0.644
** MAT ^+^**	X	0.478	0.884

**Abbreviations:** AFW, affected wrist side; BMN, bifid median nerve; EMG, electromioneurography; FS, frequency of symptoms; MAT, median artery thrombosis; TMA, thenar muscle atrophy; PT, provocative tests. **Notes:** *Student t-test; ^+^ Chi-square test.

## Discussion

The CTS is a well-known disease, but the association between the persistent median artery remains a source for discussion. The other studies and researchers have few patients, and there were no prospective studies. Cadaveric studies help to understand the median artery's epidemiology and anatomic distribution, but the CTS is still hard to understand. There were no reports about how this condition behaved and how many persistent median arteries would evolve as such, becoming the persistent median artery CTS (PMA-CTS). Diabetic and rheumatoid patients were excluded because diabetic mononeuropathy and rheumatoid features could overlap the PMA-CTS.

We know that most of the studies explain intermittent symptoms of PMA-CTS, but there is no established cutoff in the current literature. Considering our data reports, all the patients recorded intermittent nocturnal pain and numbness, worsening during summer or hot days and alleviating in the winter or cold days. Furthermore, 18 patients complained of worsening symptoms after 2 years. At this time, the intermittent symptoms become continuous. The other 7 patients searched for medical care after 3 years because of the continuous symptoms. At the first evaluation, all the patients presented a provocative positive test (Durkan and Phalen), but only 7 presented numbness during the day of the physical evaluation before testing.

Regarding the association between the persistent median artery and CTS, Barfred et al. 13 observed two distinct groups. The first group had typical symptoms, with insidious onset and chronic evolution, and presented as a surgical finding with a patent persistent median artery. The second group had symptoms of abrupt onset, with intense pain and paresthesia, presenting with a persistent MAT during the surgical procedure. 2 13

It is essential to be aware of persistent MAT as a differential diagnosis for CTS since it usually progresses with EMG without alterations, considering that the nature of aggression to the nerve, axonal degenerations will not be present initially. 1 2 7

The distribution of persistent median artery is an uncommon abnormality, which has a high incidence of association with an incomplete palmar arch, suggesting that it should be preserved to prevent symptoms of ischemia in the fingers. 7 14 15 16 17

Some studies suggest that sudden onset of neurological symptoms due to compression of the median nerve at the level of the carpal tunnel, the sensation of cold and edema must be treated as clinical warnings and should lead to the performance of ultrasound with Doppler color of the arteries of the upper limbs. 14 A computed tomography (CT) angiography can show more details with higher precision, defining the dominance of the median artery in hand perfusion, thus helping better decision-making. 14

Furthermore, some studies advocate ultrasound evaluation before the release of the carpal tunnel to evaluate the local anatomy and to observe the presence of persistent median artery. The tourniquet leads to the emptying of the vessels or damage, increasing the risk of sequelae and recurrence of the condition. 15 16 17 18 19

Gassner et al. 16 described a classification of the persistent median artery and the median nerve as normal, high division, and BMN. This division correlates the localization of the persistent median artery to the median nerve in the carpal tunnel (ulnar, radial, or intermediary). The normal median nerve has an ulnar-sided persistent median artery, while the highly divided or bifid nerve has an intermediate (central) one. 16 19 20 21

A persistent median artery was associated with the BMN in 76% of the cases, compared to other studies that presented about 63% of correlation. 12 18 Regarding the mean diameter of this artery, it was similar to that found by the same author, being 1.4 mm (0.9–4.0) against 1.3 mm (0.8–2.5 mm). 3 7

Despite the results presented, our study has few cases, and they were not submitted to decompression in the same period since the beginning of the symptoms. Given this, the statistical difference presented should be carefully analyzed. Other reports explain that arterial thrombosis worsens the symptoms. In cases of late-onset symptoms or late search for medical care, EMG abnormalities in the provocative tests are expected. The late search for medical care and the variables TMA, frequency of symptoms, and arterial thrombosis could be misunderstood. We believe that the relationship between age and total duration of the symptoms is due to CTS being uncommon in younger patients. When a younger patient complains of CTS symptoms, ultrasonography is advised. Thus, surgical intervention could be performed earlier, helping patients have fewer symptoms and complications.

## Conclusion

We conclude that, although uncommon, the persistent median artery should be considered a differential diagnosis for CTS. Ultrasonography is a reliable method to predict carpal tunnel anatomy. The late onset of symptoms could influence artery thrombosis and worsen the symptoms.
