# Supplementary material for: Persistent Median Artery and Carpal Tunnel Syndrome: A Retrospective Study
Source: Rev Bras Ortop (Sao Paulo). 2024 May 13;59(6):e895–900. doi: 10.1055/s-0044-1785657 (PMC11663071; doi:10.1055/s-0044-1785657)
Supplement: Related texts

# Research article: Artéria mediana persistente e síndrome do túnel do carpo: Um estudo retrospectivo

**Keywords:** artéria mediana, nervo mediano, neuropatia mediana, síndrome do túnel carpal

## Abstract

**Objetivo** Este é um estudo retrospectivo acerca da persistência da artéria mediana associada à síndrome do túnel do carpo (STC).

**Métodos** Este é um estudo retrospectivo da persistência da artéria mediana e STC. Os critérios de exclusão foram pacientes que não apresentavam artéria mediana persistente, aqueles que eram diabéticos ou reumatoides e os que decidiram não realizar a cirurgia. Apenas 25 pacientes foram elegíveis para este estudo retrospectivo.

**Resultados** A trombose da artéria mediana apresentou diferenças estatísticas considerando as variáveis sexo ( *p* = 0,009), achados eletroneuromiográficos ( *p* = 0,021), profissão ( *p* = 0,066) e “duração total desde o início dos sintomas” ( *p* = 0,055). A atrofia da musculatura tenar não apresentou diferenças estatísticas à comparação das variáveis. O nervo mediano bífido apresentou diferenças estatísticas em comparação aos testes provocativos ( *p* = 0,013), frequência dos sintomas ( *p* = 0,001) e idade ( *p* = 0,028).

**Conclusão** Embora incomum, a persistência da artéria mediana deve ser considerada um diagnóstico diferencial da STC. A ultrassonografia é um método confiável para prever a anatomia do túnel do carpo. O início tardio e os sintomas podem influenciar a trombose arterial e piorar os sintomas.

## Introdução

A síndrome do túnel do carpo (STC) é um distúrbio compressivo do nervo mediano ao nível do punho que acomete aproximadamente 4% da população geral e é a neuropatia compressiva mais comum do membro superior. 1 2 Esta doença foi inicialmente descrita como uma compressão causada por uma fratura distal do rádio por Paget em 1854. 3 4 5 O nervo mediano progride para o nível do punho adjacente a nove tendões no túnel do carpo, um canal osteofibroso inextensível. Os pacientes queixam-se de dor e parestesia com piora à noite. A fraqueza dos músculos tenares é comum e pode evoluir para atrofia desta musculatura. 5 6

Diversas causas podem comprimir o nervo mediano, sejam elas idiopáticas ou secundárias. 6 Entre as causas secundárias, estão alterações endócrino-metabólicas (gravidez, diabetes), fraturas e luxações, tumores (gânglios, lipomas), causas sistêmicas (reumatoides), variações anatômicas e a persistência da artéria mediana. 2 6 7

A artéria mediana é observada em cerca de 0,9 a 16% da população. 7 Esta artéria é observada no período embrionário, sendo a corrente sanguínea dominante na mão do embrião, auxiliando o desenvolvimento das artérias do membro superior e involuindo a partir da 8ª semana da gestação. Nesta fase, inicia-se a formação das artérias ulnar e radial, que se tornarão o principal suprimento vascular do território distal do membro superior. 7 8 9

A persistência da artéria mediana no período fetal poderia contribuir para o desenvolvimento do arco palmar superficial, que pode ser mais exuberante que o habitual. Ao trafegar pelo túnel do carpo junto com o nervo mediano, embora rara, pode desencadear a STC. 10

A persistência da artéria mediana pode ocorrer em dois padrões: antebraquial e palmar. 11 No primeiro, a artéria mediana termina como um ramo muscular, não atingindo o punho não acometido. No segundo tipo, a artéria mediana estende-se até o território palmar, atravessando o túnel do carpo, participando algumas vezes da formação do arco palmar superficial e tornando-se uma possível causa da STC. 2 8 11 12

## Métodos

Este estudo foi aprovado pelo comitê de ética em pesquisa (CAAE-45542621.2.0000.5440) de nossa instituição. Os pacientes e suas famílias foram informados que os dados do caso seriam submetidos para publicação e deram seu consentimento.

O estudo retrospectivo reviu prontuários de pacientes com STC associada à persistência da artéria mediana e submetidos à cirurgia de descompressão em um hospital terciário.

No total, 1.276 pacientes com STC foram submetidos à descompressão entre 2012 e 2020. Os critérios de inclusão foram pacientes que apresentaram persistência da artéria mediana e STC de 2012 a 2020, gerando uma amostra de 38 indivíduos. Os critérios de exclusão foram pacientes que não apresentavam persistência da artéria mediana, tinham diabetes e/ou doenças reumáticas e que decidiram não realizar a cirurgia. Apenas 25 pacientes foram elegíveis para este estudo retrospectivo. Utilizamos os dados presentes nos prontuários e exames de imagem, como ultrassonografia e ressonância magnética ( Fig. 1 ).

**Fig. 1:** Ultrassonografia. **A:** Nervo mediano (seta, círculo). **B:** Artéria mediana persistente (seta). **C:** Nervo mediano bífido (cabeças de setas). **D, E, F:** Ressonância magnética. **D:** Nervo mediano (seta). **E:** Nervo mediano bífido (pontas de seta). **F:** Artéria mediana persistente (seta).

A média de idade dos pacientes foi de 41 anos (intervalo de 18 a 73 anos), sendo 17 mulheres e oito homens. Vinte e quatro pacientes eram destros. A profissão e as variáveis dos pacientes são apresentadas na Tabela 1 . Os testes provocativos (Durkan e Phalen) foram realizados em todos os pacientes durante a avaliação física. Onze pacientes apresentaram dormência durante a avaliação física. Cinco pacientes relataram sintomas intermitentes e, em 20 indivíduos, os sintomas eram contínuos. Todos os pacientes queixavam-se de fraqueza; entretanto, apenas quatro pacientes apresentaram atrofia tenar (AMT). O protocolo pré-operatório incluiu ultrassonografia (Doppler) para mensuração do túnel do carpo e do nervo mediano. A eletroneuromiografia (EMG) foi realizada em todos os pacientes.

**Tabela 1:** Casos e variáveis

	Sexo	Idade (anos)	Etnia	Dominância	LPA	Profissão	Comorbidades	EMG	TP	FS	AMT	NMB	TAM	DSAC (meses)
**Caso 1**	F	60	AA	D	D	Cozinha	HAS	S	DP	C	N	S	N	50
**Caso 2**	F	36	CC	D	D	Manicure	N	S	DPD	C	N	S	N	49
**Caso 3**	F	40	AA	D	D	ED	HAS	N	DPD	C	S	S	N	36
**Caso 4**	F	40	AA	D	E	ED	HAS	N	DPD	C	N	S	N	52
**Caso 5**	F	28	AA	D	D	ED	N	N	DPD	C	N	S	N	216
**Caso 6**	F	26	CC	D	E	ED	N	N	DPD	C	N	S	N	204
**Caso 7**	F	31	CC	D	D	ED	N	N	DPD	C	S	S	N	130
**Caso 8**	M	45	CC	D	D	SG	HAS	N	DPD	C	N	S	N	133
**Caso 9**	M	49	CC	E	E	ED	HAS	N	DPD	C	N	S	N	131
**Caso 10**	F	38	AA	D	E	Motorista	N	N	DPD	C	S	S	N	118
**Caso 11**	F	33	CC	D	D	SG	N	N	DPD	C	N	S	N	83
**Caso 12**	F	35	CC	D	E	ED	N	N	DPD	C	N	S	N	72
**Caso 13**	M	44	CC	D	D	Enfermeiro	N	N	DP	C	S	S	S	41
**Caso 14**	F	26	AA	D	E	Enfermeira	N	S	DP	C	N	S	S	42
**Caso 15**	M	18	CC	D	D	SG	N	S	DP	C	N	S	S	33
**Caso 16**	M	45	CC	D	D	Empresário	N	S	DP	C	N	S	S	48
**Caso 17**	M	35	AA	D	E	Empresário	N	S	DP	C	N	S	S	47
**Caso 18**	M	44	CC	D	D	Professor	N	S	DP	C	N	S	S	52
**Caso 19**	F	38	CC	D	D	Professora	N	N	DP	C	N	N	S	55
**Caso 20**	F	64	CC	D	D	Empresário	N	S	DP	I	N	N	N	51
**Caso 21**	F	45	CC	D	D	Cozinha	N	N	DP	I	N	N	N	49
**Caso 22**	F	30	AA	D	E	SG	N	N	DP	I	N	N	N	47
**Caso 23**	F	54	CC	D	D	Costureira	HAS	N	DP	I	N	N	N	39
**Caso 24**	F	48	AA	D	D	Cozinha	HAS	N	DP	I	N	S	N	44
**Caso 25**	M	73	CC	D	E	Professor	HAS	S	DP	C	N	N	N	91

**Abreviações:** AA, afro-americana; AMT, atrofia da musculatura tenar; C, contínua; CC, caucasiana; D, direito; DP, Durkan-Phalen; DPD, Durkan-Phalen-Dormência; DSAC, duração dos sintomas até a cirurgia; E, esquerdo; ED, empregado(a) doméstico(a); EMG, eletroneuromiografia; F, feminino; FS, frequência de sintomas; HAS, hipertensão arterial sistêmica; I, intermitente; LPA, lado do punho acometido; M, masculino; N, não; NMB, nervo mediano bífido; S, sim; SG, serviços gerais; TAM, trombose da artéria mediana; TP, testes provocativos.

Os pacientes foram submetidos à descompressão cirúrgica do nervo mediano ao nível do punho. A cirurgia foi realizada em 17 punhos direitos e oito punhos esquerdos ( Fig. 2 ). Havia 19 pacientes apresentando nervo mediano bífido (NMB) à ultrassonografia. A avaliação cirúrgica revelou sete casos de trombose da artéria mediana (TAM). Nenhuma complicação foi relatada (infecção, complicações da ferida, recidiva). O acompanhamento médio foi de 76,5 meses (intervalo de 33 a 216), sem relatos de recidiva.

**Fig. 2:** Aspecto operatório da artéria mediana persistente (seta).

O teste t de Student e o teste qui-quadrado foram utilizados para análise das variáveis. Um valor de *p* bicaudal inferior a 0,05 foi considerado estatisticamente significativo. Todas as análises foram realizadas no Statistical Package Social Sciences (SPSS, IBM Corp., Armonk, NY, EUA) for Os X, versão 22.0.0.

## Resultados

Não houve diferenças estatísticas entre as variáveis dominância (qui-quadrado: *p* = 0,524; Fisher: *p* = 1,000), lado do punho afetado (qui-quadrado: *p* = 0,819; Fisher: *p* = 1,000), etnia (qui-quadrado: *p* = 0,856; Fisher: *p* = 1,001), NMB (qui-quadrado: *p* = 0,478; Fisher: *p* = 0,637), AMT (qui-quadrado: *p* = 0,884; Fisher: *p* = 1,000), frequência dos sintomas (qui-quadrado: *p* = 0,119; Fisher: *p* = 0,274) e TAM.

Houve diferenças estatísticas na TAM considerando as variáveis sexo (qui-quadrado: *p* = 0,009; Fisher: *p* = 0,023), achados EMGs (qui-quadrado: *p* = 0,021; Fisher: *p* = 0,061), comorbidades (qui-quadrado: *p* = 0,032; Fisher: *p* = 0,057), teste provocativo (qui-quadrado: *p* = 0,006; Fisher: *p* = 0,008) e profissão (qui-quadrado: *p* = 0,066), sendo os empregados domésticos os mais propensos a ter TAM.

Houve diferenças estatísticas entre as variáveis “duração total desde o início dos sintomas” (ANOVA: *p* = 0,055; Levene: 0,003) e TAM. Não houve diferenças estatísticas nas variáveis idade (ANOVA: *p* = 0,197; Levene: 0,461) e TAM.

Não houve diferenças estatísticas entre as variáveis sexo (qui-quadrado: *p* = 0,743; Fisher: *p* = 1,000), dominância (qui-quadrado: *p* = 0,656; Fisher: *p* = 1,000), lado do punho afetado (qui-quadrado: *p* = 0,743; Fisher: *p* = 1,000), etnia (qui-quadrado: *p* = 0,119; Fisher: *p* = 0,292), profissão (qui-quadrado: *p* = 0,222), comorbidades (qui-quadrado: *p* = 0,743; Fisher: *p* = 1,000), achados EMGs (qui-quadrado: *p* = 0,102; Fisher: *p* = 0,261), testes provocativos (qui-quadrado: *p* = 0,173; Fisher: *p* = 0,303), frequência dos sintomas (qui-quadrado: *p* = 0,275; Fisher: *p* = 0,549), NMB (qui-quadrado: *p* = 0,220; Fisher: *p* = 0,540) e AMT. Não houve diferenças estatísticas entre as variáveis “duração total desde o início dos sintomas” (ANOVA: *p* = 0,844; Levene: 0,811) ou idade (ANOVA: *p* = 0,644; Levene: 0,111) e AMT.

Não houve diferenças estatísticas entre as variáveis sexo (qui-quadrado: *p* = 0,356; Fisher: *p* = 0,624), dominância (qui-quadrado: *p* = 0,566; Fisher: *p* = 1,000), lado do punho afetado (qui-quadrado: *p* = 0,356; Fisher: *p* = 0,624), etnia (qui-quadrado: *p* = 0,181; Fisher: *p* = 0,345), profissão (qui-quadrado: *p* = 0,270), comorbidades (qui-quadrado: *p* = 0,936; Fisher: *p* = 1,014), achados EMGs (qui-quadrado: *p* = 0,876; Fisher: *p* = 1,000). Houve diferenças estatísticas entre as variáveis testes provocativos (qui-quadrado: *p* = 0,013; Fisher: *p* = 0,020), frequência dos sintomas (qui-quadrado: *p* = 0,001; Fisher: *p* = 0,005) e NMB. Não houve diferenças estatísticas entre as variáveis “duração total desde o início dos sintomas” (ANOVA: *p* = 0,251; Levene: 0,018) e NMB. Contudo, houve diferença estatística na variável idade (ANOVA: *p* = 0,028; Levene: 0,089) e NMB.

A Tabela 2 mostra os resultados das variáveis. Todos os estudos ultrassonográficos e os achados operatórios (NMB e artéria mediana persistente) foram estatisticamente relevantes (qui-quadrado: *p* = 0,001).

**Tabela 2:** Resultados das variáveis estudadas e trombose da artéria mediana, nervo mediano bífido e atrofia da musculatura tenar

	TAM	NMB	AMT
** Sexo ^+^**	0,009	0,356	0,743
** Dominância ^+^**	0,524	0,566	0,656
** LPA ^+^**	0,819	0,356	0,743
** Etnia ^+^**	0,856	0,181	0,119
** NMB ^+^**	0,478	X	0,220
** FS ^+^**	0,119	0,001	0,275
** AMT ^+^**	0,884	0,220	X
** Achados à EMG ^+^**	0,021	0,876	0,102
** Profissão ^+^**	0,066	0,270	0,222
** Comorbidades ^+^**	0,032	0,936	0,743
** TP ^+^**	0,006	0,013	0,173
Duração total desde o início dos sintomas*	0,055	0,251	0,844
Idade*	0,197	0,028	0,644
** TAM ^+^**	X	0,478	0,884

**Abreviações:** AMT, atrofia da musculatura tenar; EMG, eletroneuromiografia; FS, frequência de sintomas; LPA, lado do punho acometido; NMB, nervo mediano bífido; TAM, trombose da artéria mediana; TP, testes provocativos. **Notas:** *Teste t de Student; ^+^ teste qui-quadrado.

## Discussão

A STC é uma doença bem conhecida, mas sua associação com a artéria mediana persistente continua a ser discutida. Os demais estudos e pesquisadores têm poucos pacientes e não houve estudos prospectivos. Estudos cadavéricos auxiliam a compreensão da epidemiologia e distribuição anatômica da artéria mediana, mas a STC ainda é difícil de compreender. Não houve relatos sobre o comportamento da síndrome e quantas artérias medianas persistentes evoluiriam para tal, tornando-se a STC com artéria mediana persistente (STC-AMP). Os pacientes diabéticos e reumatoides foram excluídos porque a mononeuropatia diabética e as características reumatoides poderiam se sobrepor aos da síndrome.

Sabemos que a maioria dos estudos explica os sintomas intermitentes da STC-AMP, mas a literatura atual não registra um ponto de corte. Considerando nossos dados, todos os pacientes relataram dor noturna intermitente e dormência, piorando durante o verão ou dias quentes e melhorando no inverno ou dias frios. Além disso, 18 pacientes queixaram-se de piora dos sintomas após 2 anos. Neste momento, os sintomas intermitentes tornam-se contínuos. Os outros 7 pacientes procuraram atendimento médico após 3 anos devido aos sintomas contínuos. Na primeira avaliação, todos os pacientes apresentaram teste provocativo positivo (Durkan e Phalen), mas apenas 7 apresentaram dormência durante o dia da avaliação física antes do teste.

Quanto à associação entre a persistência da artéria mediana e a STC, Barfred et al. 13 observaram dois grupos distintos. O primeiro apresentou sintomas típicos, de início insidioso e evolução crônica, com achado cirúrgico de artéria mediana persistente e patente. O segundo grupo apresentou sintomas de início abrupto, com dor intensa e parestesia, com observação de TAM persistente durante o procedimento cirúrgico. 2 13

É fundamental estar atento à TAM persistente como diagnóstico diferencial da STC, pois geralmente a EMG não revela alterações, já que, devido à natureza da agressão ao nervo, não há degenerações axonais iniciais. 1 2 7

A distribuição da artéria mediana persistente é uma anomalia incomum, com alta incidência de associação com o arco palmar incompleto, sugerindo que deve ser preservada para prevenir sintomas de isquemia nos dedos. 7 14 15 16 17

Alguns estudos sugerem que os sintomas neurológicos de início súbito de compressão do nervo mediano à altura do túnel do carpo, a sensação de frio e o edema devem ser tratados como alertas clínicos, levando à realização de ultrassonografia com Doppler colorido das artérias dos membros superiores. 14 A angiotomografia pode mostrar mais detalhes com maior precisão, determinando a dominância da artéria mediana na perfusão da mão e auxiliando a melhor tomada de decisão. 14

Além disso, alguns estudos defendem a avaliação ultrassonográfica antes da descompressão do túnel do carpo para avaliação da anatomia local e observação da presença da artéria mediana persistente. O torniquete provoca esvaziamento dos vasos ou danos, aumentando o risco de sequelas e recidiva do quadro. 15 16 17 18 19

Gassner et al. 16 descreveram uma classificação da artéria mediana persistente e do nervo mediano como normal, em divisão alta e NMB. Esta divisão correlaciona a localização da artéria mediana persistente ao nervo mediano no túnel do carpo (ulnar, radial ou intermediário). O nervo mediano normal tem uma artéria mediana persistente no lado ulnar, enquanto o nervo com divisão alta ou bífido é acompanhado por uma artéria intermediária (central). 16 19 20 21

A artéria mediana persistente foi associada ao NMB em 76% dos casos, enquanto outros estudos apresentaram cerca de 63% de correlação. 12 18 O diâmetro médio desta artéria foi semelhante ao encontrado pelo mesmo autor, sendo de 1,4 mm (0,9–4,0) contra 1,3 mm (0,8–2,5 mm). 3 7

Apesar dos resultados apresentados, nosso estudo possui poucos casos, que não foram submetidos à descompressão no mesmo período desde o início dos sintomas. Diante disso, a diferença estatística apresentada deve ser analisada com cautela. Outros relatos explicam que a trombose arterial piora os sintomas. Nos casos de início tardio dos sintomas ou procura tardia por atendimento médico, espera-se a observação de EMG alteradas nos testes provocativos. A procura tardia por atendimento médico e as variáveis AMT, frequência de sintomas e trombose arterial podem ser mal interpretadas. Acreditamos que a relação entre idade e duração total dos sintomas se deve ao fato de a STC ser incomum em pacientes mais jovens. Quando um paciente mais jovem se queixa de sintomas de STC, a ultrassonografia é recomendada. Assim, a intervenção cirúrgica poderia ser realizada antes, ajudando o paciente a ter menos sintomas e complicações.

## Conclusão

Concluímos que, embora incomum, a persistência da artéria mediana deve ser considerada um diagnóstico diferencial para a STC. A ultrassonografia é um método confiável para prever a anatomia do túnel do carpo. O início tardio dos sintomas pode influenciar a TAM e piorar os sintomas.
